# Small but Terrible: Utilizing Chitosan-Based Nanoparticles as Drug Carriers to Treat Tuberculosis in the Philippines

**DOI:** 10.3389/fphar.2021.752107

**Published:** 2021-10-06

**Authors:** Janela Rose Agpangan Limocon, Lyka Marie Cawaling Madalag, Paulyn Jade Balungcas Reliquias, Janina Veana Soriano Tionko, Jamie Ledesma Fermin, Shaira Limson Kee, Myles Joshua Toledo Tan, Maria J-len Juesna Jonco, Ma. Junallie Fuentebella Pomperada

**Affiliations:** ^1^ College of Nursing, University of St. La Salle, Bacolod, Philippine; ^2^ Department of Electronics Engineering, University of St. La Salle, Bacolod, Philippines; ^3^ Department of Natural Sciences, University of St. La Salle, Bacolod, Philippines; ^4^ Department of Chemical Engineering, University of St. La Salle, Bacolod, Philippines; ^5^ Department of Materials Engineering, University of St. La Salle, Bacolod, Philippines

**Keywords:** tuberculosis, Chitosan, nanoparticles, Philippines, multidrug-resistant tuberculosis, nanotechnology, drug carriers, drug delivery system

## Introduction

Tuberculosis (TB), a contagious disease caused by *Mycobacterium tuberculosis* (Mtb), is one of the major reasons for increasing mortality in developing nations (Kirtane et al., 2021). According to the World Health Organization (WHO), the Philippines ranked first in Asia in terms of TB cases in 2019 with 554 cases per 100,000 population diagnosed with the disease. ∼74 deaths due to TB are tallied daily, making it one of the 10 leading causes of mortality in the country. Current solutions for this disease consist of the standard treatments for 6 months using drugs like rifampicin (RIF), isoniazid (INH), ethambutol, and pyrazinamide. The treatment is complemented with a two-month continuation phase using INH and RIF. Treatment duration varies depending on whether the patient is diagnosed with multidrug-resistant tuberculosis (MDR-TB), which requires him/her to comply with a longer treatment course (Nabi et al., 2020). According to the Comprehensive and Unified Policy (CUP) for TB Control in the Philippines, the National Tuberculosis Control Program (NTP) in the Philippines was established in 1954 to oversee the TB mitigation efforts (2004). Despite all measures, factors like long duration of treatment, frequent dosing, side effects, and distance to health clinics increase the likelihood of patient non-adherence. These result in greater risk of acquiring MDR-TB (Kirtane et al., 2021; Zegeye et al., 2019). As reported by the Department of Health, 2020, the treatment success rate of drug susceptible forms of TB reached 92%, surpassing the target rate of 90% by the end of the year. However, TB case detection rate and treatment success rate were reported to be 32% (<62% target) and 49% (<75% target), respectively. As reported by the CUP for TB Control in the Philippines, it has been 72 years since the first usage of streptomycin for TB. Despite the availability of viable anti-TB medications in the country, they have not been fully successful as an intervention. Hence, there is a need to advance TB treatment by supporting other mitigation strategies that could benefit the Philippines.

In 2016, Garg et al. wrote “Inhalable chitosan nanoparticles as antitubercular drug carriers for effective treatment of tuberculosis,” which presented a potential enhancement to TB treatment. They showed that drug-loaded CNPs are efficient transporters for alveolar macrophage targeting. This enhanced formulation was shown to bring about a substantial reduction in the amount of bacilli in the lungs and a decrease in cytotoxicity when compared with free drugs. These positive results could mean improvements to the much-needed TB drug delivery in the Philippines. Chitosan has also shown great potential for drug advancements that come in various forms from nanoparticles to conjugates, tablets, and vaccines (Cheung et al., 2015). Chitosan is among the commonly used polymers as it can be prepared in moderate conditions, has similar stiffness as the body, appropriate biodegradability, and low crystallinity that can be optimally combined with many kinds of drugs (Rajan and Raj, 2012). Other unique properties of chitosan that make it medically valuable are biocompatibility, low immunogenicity, and its biological activities. Among these properties, the most crucial is the solubility of chitosan in the most acidic organic solution. When the pH is less than 6.5, nanoparticles are easily formed through crosslinking with agents like glutaraldehyde, polyaspartic acid sodium salt, and tripolyphosphate (Praphakar et al., 2017). CNPs are considered to be great drug carriers due to their excellent biodegradability and biocompatibility (Cheung et al., 2015), making them safe and non-immunogenic to body tissues. They also have antimicrobial, wound healing, and muco-adhesion properties that make them good drug carriers (Jhaveri et al., 2021). According to Rajan and Raj in 2012, the mechanism behind the formation of the nanoparticle form of the chitosan-based drug carrier is the ionic action between the positively charged chitosan solution and the negatively charged tripolyphosphate (TPP) solution. The reported method for preparing chitosan nanoparticles involves the dissolution of Chitosan, TPP, and RIF in solutions such as aqueous acetic acid, distilled water, and TPP solution, respectively. The TPP solution containing the dissolved combination drugs was combined with the chitosan solution under magnetic stirring at room temperature. From this method, various amounts of chitosan can be produced instantaneously. Polyethylene glycol (PEG) was gently added to a suitable quantity of various percentage encapsulated chitosan-RIF. Also, Magnetic agitation at 25°C for 90 min followed in order to bind the chitosan. Following that, drug loading capacity, encapsulation, and an *in vitro* drug release evaluation were examined. The poor solubility of RIF in aqueous media was improved by the surface coating of chitosan, a hydrophilic polymer, and PEG. PEG binding with chitosan-RIF changed the character and nanoparticle surface and caused a slight increase in particle size, while the drug encapsulation also increased. The chitosan and chitosan-PEG nanoparticles overcome the limitations of liposomes, the first clinical carrier, to efficiently encapsulate hydrophobic drugs into a biocarrier and control the release of drugs, thereby making it a promising system for delivering treatment of TB.

In the Philippines, studies on chitosan nanotechnology have been explored with financial support from different government agencies, such as the Commission on Higher Education (CHED), Department of Science and Technology–Philippine Council for Industry, Energy and Emerging Technology Research and Development (DOST-PCIEERD), and DOST–Philippine Council for Health Research and Development (DOST-PCHRD). The development priorities of these agencies include the enhancement of healthcare, among others, giving hope to future research into CNPs that could benefit the Philippines and other countries where TB is prevalent.

### The Effectiveness of Nanotechnology for Drug Delivery Systems

The development of nanotechnology has improved the delivery of drugs for the treatment of numerous diseases such as cancer by enhancing targeted delivery to minimize the progression of disease (Gmeiner and Ghosh, 2013; Saeedi et al., 2019). However, it is difficult for preclinical studies to be translated into clinical trials because of multiple factors such as economics and ethics, among many others (Zhang et al., 2018). Still, there have been successful studies that have led to improved drug delivery and that have been approved by the United States (US) Food and Drug Administration (FDA), such as Afrezza®- an inhalable drug indicated for type 1 diabetes mellitus (Muralidharan et al., 2015).

Moreover, inhalable CNP was found to possess better target site-specific and sustained drug delivery, than free drugs (Garg et al., 2019). Besides this, nanoparticles as drug carriers are engineered to obey the Three Rs of drug delivery: right targeted area, right timing, and right loaded dose (Zhang et al., 2018). With this, they efficiently retain the drug in the specific area, allowing proper absorption and resulting in a more effective process. To date, more than 50 clinical formulations have been made available in the market to address various ailments. Worthy of emphasis is the success of nanoformulated Onpattro^®^ as a novel therapeutic for patients diagnosed with transthyretin amyloidosis (Germain et al., 2020). Furthermore, the success rate of using nanomedicine formulations for cancer treatment have been found to be greater than conventional ones like Paclitaxel (PTX) and Docetaxel (DTX) intended for treating malignancies (Liu et al., 2011; Selvarathinam et al., 2021). These successes have led countries like the US and China to allocate sizable fractions of their budgets for nanotechnology research and manufacturing to address other pressing issues in medicine such as in areas of cancer detection and treatment (Gao et al., 2015).

### The Potential of Chitosan for Tuberculosis Treatment

Chitosan has multiple properties that can aid in the treatment of TB. For instance, it is known for its biocompatibility and biodegradability which promotes good adhesion to mucosal surfaces. It also increases the absorption of vaccines and drugs, lengthens the duration of therapeutic effects for drug effectivity and facilitates drug delivery to specific body sites (Khademi et al., 2018; Garg et al., 2019). Moreover, chitosan exhibits a broad spectrum of antimicrobial activity against fungi, yeast, and gram-positive bacteria (Benhabiles et al., 2012; Raafat and Sahl., 2009). For instance, an assessment of *in vitro Mycobacterium* bovis susceptibility by Cunha et al. (2019) found that using chitosan microparticles impedes Mycobacterial growth by 95–100%. After an initial evaluation, results showed that chitosan microparticles exhibited a potent ability to activate and sustain macrophage uptake by up to 99.9%. Chitosan is also used to prevent bacterial colonization (Jhaveri et al., 2021). This means that it could potentially reduce Mtb colonization, as well.

There are also concerns regarding much longer treatment courses required of patients with MDR-TB (Nabi et al., 2020). The extended treatment length results in poor patient adherence (Tan et al., 2020) and risk of relapse (Pawde et al., 2020). Fortunately, chitosan can serve as a potential carrier for drugs such as INH and RIF (Garg et al., 2019). Moreover, CNP inhalation can effectively treat TB while bypassing the gastrointestinal tract, thereby preventing issues such as poor solubility which decreases drug absorption. This lessens the required drug dose and helps promote patient compliance. Garg et al., 2019 experimented on mice injected with Mtb to evaluate the effectiveness of inhaled CNPs with smooth spherical surfaces that freely capture alveolar macrophages. This treatment exhibited responses to Mtb that were notably more efficient than free drugs. Mycobacterial colony forming units in the lungs of the Mtb-infected mice in the antitubercular drug-loaded CNP group decreased the amount of bacilli from 1.23 $log_{10}$ CFU to 0.19 $log_{10}$ CFU, while the RIF-INH group saw a decrease in the amount of bacilli from 5.99 $log_{10}$ CFU to 1.34 $log_{10}$ CFU. With these remarkable results, Chitosan has great potential in treating TB as an inhalable therapy for pulmonary TB.

### Chitosan-Based Nanoparticles as Drug Carriers Versus Conventional Treatments

Though considered an efficacious course of treatment, the conventional anti-tubercular drug treatment plan is associated with several problems. These include lack of metabolic stability, low membrane permeability, low solubility, high-loaded dosage forms and hepatotoxicity (Nabi et al., 2020). Traditional TB treatment also entails precise dosages and frequencies, and lengthy treatment periods that lead to patient noncompliance (Tan et al., 2020). So, despite the availability of current antibiotics, there is still a large demand for other treatment options (Nabi et al., 2020). This demand gave rise to solutions that rely on nanotechnology, such as CNPs. Nanoparticles are colloidal particle carriers of drugs made up of polymers in the nanoscopic scale that are either synthetic or natural (Garg et al., 2019). Their molecular topologies give rise to various pharmacologic properties that allow them to release drugs at controlled and sustained rates, improve drug solubility, lower toxicity, enhance efficacy, and target particular areas nasally, transdermally, corneally, gastrointestinally, or intravenously. Given their nanosize and greater surface area, they can pass cell barriers by endocytosis or receptor-mediated transcytosis, resulting in better molecular release inside cells (Iacob et al., 2021). These properties allow them to overcome obstacles faced by conventional treatment and as a result, improve patient compliance, therapeutic effects, and overall clinical outcomes (Jhaveri et al., 2021).

Chitosan is a great natural polymer because it has enormous potential in the development of nanocarrier systems (Jhaveri et al., 2021). Chitosan has also been shown to be biodegradable, nonimmunogenic, biocompatible, non-toxic, and bioadhesive, making it ideal for use as a drug delivery system (Iacob et al., 2021). Chitosan shows remarkable biocompatibility because of its similarities with glycosaminoglycans found in extracellular matrices of the human body. Moreover, by means of the cleavage of glycosidic bonds of chitosan, it is quickly broken down by chitinases, lysozyme, and microbes in the colon (Jhaveri et al., 2021). Physical and chemical modifications can also be made to enhance specific properties such as solubility, steadiness, and mucoadhesion, allowing chitosan to efficiently serve its purpose and widen its applications (Mohammed et al., 2017). And very importantly, chitosan is considered safe for human use, as affirmed by the US FDA (Garg et al., 2019). All these reasons make chitosan a good choice for drug delivery through various administration routes, e.g., oral, nasal, buccal, pulmonary, and vaginal (Mohammed et al., 2017).

### The Feasibility of the Use of Chitosan-Based Nanoparticles as Drug Delivery Systems in the Philippines

Nanotechnology and its applications have been some of the most promising research and development (R and D) areas of the decade (Wong et al., 2013; Conde, 2015; Zakeri and Lu, 2015). However, nanotechnology demands large amounts of funding, similar in size to the space program of the US government (DeFrancesco, 2003). The Philippines, spearheaded by DOST-PCIEERD, has begun implementing a roadmap that focuses on genomics and nanotechnology to address multifaceted problems in the country (Anonas, 2012). Despite the roadmap, the country’s R and D budget is still below the 1% GDP allocation suggested by the United Nations Educational, Scientific and Cultural Organization (UNESCO) for developing countries. Moreover, expenditures for R and D in the Philippines appear to have stagnated when compared with other ASEAN states (Albert et al., 2015).

Recently, however, DOST-PCIEERD funded Filipino researchers to develop innovations in nanotechnology, to enhance COVID-19 response. Fortunately, the Philippines, being an archipelago, is rich in aquatic resources. An estimate of >250,000 metric tons of carapace waste comes from the Philippines (Cadano et al., 2020). The exoskeletons of seafood waste are excellent sources of chitin that can be used to produce chitosan through a deacetylation reaction (White et al., 1979; De Queiroz Antonino et al., 2017; Younes and Rinaudo, 2015). The vast availability of seafood waste in the Philippines could form the basis for the development of an entire industry focused on drug delivery systems that would solve the TB crisis in the country and throughout the globe. Also, further research about the resources of the Philippines and their use for nanotechnology may strengthen healthcare in the country, and maybe globally as well.

To date, only a total of 41 nanotechnology projects funded by the DOST-PCIEERD have been completed, while five are ongoing. For instance, quercetin nanoliposomes from rice bran phospholipids have been found to enhance phytochemical properties and to be efficient oral drug delivery vehicles (Rodriguez et al., 2018). Another notable example is a 0.005% latanoprost eyedrop formulated in the country from hyaluronic acid-chitosan-latanoprost link nanoparticles that have been found to reduce intraocular pressure and enhance target-specific drug concentrations more potently than commercially available latanoprost solutions (Rubenicia et al., 2021).

Efforts to investigate CNP properties to enhance drug delivery systems have been carried out throughout the years. The Philippines, along with Egypt and 47 other countries, was classified as a lower-middle-income economy by the United Nations World Economic Situation and Prospects (WESP) Report in Mid-2021. Lower-middle-income countries have used nanotechnology in healthcare research. A notable example is the Cancer Nanotechnology Research Laboratory in Egypt that investigates ways to enhance drug delivery through various routes, including that of the pulmonary tract (Elzoghby, 2019). For instance, they have found a way to target lung cancer cells through inhalable nanocomposites formulated with etoposide phospholipid complexes encapsulated in albumin nanoparticles and berberine, resulting in greater efficacy in contrast with free drugs (Elgohary et al., 2018). Because of this, support for nanotechnology research in this lower-middle-income economy is strong. Clinical translations and manufacturing in Egypt are encouraged as they offer significant benefits to healthcare provision (Elzoghby, 2019). Moreover, a study by Fermin and Tan (2020) reported that there exists a strong positive relationship between various indicators of health among countries in Southeast Asia and the volume of publications written on biomedical engineering research, which includes research in biomaterials engineering for the development of drugs and drug delivery systems. Accordingly, the Philippines must establish strong support for nanotechnology research, as well, to encourage more endeavors in the improvement of drug delivery systems that could potentially streamline the process of combating TB in the country.

## Discussion

The Philippines is surrounded by large bodies of water endowed with abundant aquatic resources which is advantageous for the production of chitosan-based materials. With this, the abundance of seafood waste in the Philippines could serve as the foundation for the development of the pharmaceutical industry centered on drug delivery systems that would mitigate the country’s and the world’s TB crisis. The Philippine government can greatly utilize CNP technology as an advantage in the health sector. However, in order to attain the goal of fulfilling the reduction of TB mortality and incidence in the country, we would like to point out substantial recommendations for the government to follow. To begin with, direct grant-in-aid programs of the R and D sector focusing on nano-based drug delivery systems must be given thorough attention. The DOST-PCIEERD is the most fitting organization to advocate for chitosan-based nanotechnology. This could open opportunities for funding toward training scientists in drug delivery research.

The framework to establish industry participation could start from harnessing carapace wastes from restaurants, seafood markets and processing companies. Chitosan manufacturers could retrieve these wastes for processing, which then could be donated or sold to research institutions, specifically those inclined in nanotechnology research. Chitosan manufacturing presents opportunities for future investments for entrepreneurs nationally and globally, creating additional sources of profit that may boost economies. Both industrial and pharmaceutical research sectors must come up with agreements to properly utilize CNPs for processing into medications that can help combat MDR-TB. Carapace wastes can also prove to be a very profitable industry that could draw interest from seafood processors, entrepreneurs, and researchers who aim to convert carapace wastes into useful products that may be used in a variety of industries. Proper usage of these shell wastes not only solves the issue of their disposal, but also maximizes profit and utilization of resources.

In addition, collaboration with the government and pharmaceutical companies could be initiated to enhance drug research and manufacturing. Republic Act No. 2067 of the Philippines known as the “Science Act of 1958” is an act to integrate, coordinate, and intensify scientific and technological research and development and to foster invention; to provide funds therefor; and for other purposes. The existing provisions under this act can greatly support this industry because it can further advancements that may later aid the national goal of a TB-free Philippines. Lastly, the Philippine government should be open to new innovative approaches that will raise the standards of technology in healthcare.

This idea is still a subject for further R and D. Additional studies are needed for pre-clinical phases to be translated into clinical trials and thorough investigations are necessary for them to be publicly available. The study of Garg et al., 2015 entitled “Inhalable chitosan nanoparticles as antitubercular drug carriers for effective treatment of tuberculosis” is a well-founded reference to guide future R and D. CNP research will bring about the betterment of Philippine public health as it will be able to answer a longstanding problem in the country. It could also mean massive improvements in waste management as it utilizes seafood wastes for innovation. Also, it could translate to various benefits in the economy as it could lower healthcare costs for numerous Filipinos suffering from TB.

With this, we would like to think that with sufficient funding and proper policy-making, the use of nanotechnology could be a possible solution to TB and other health problems in the Philippines.
